# The voluntary medical male circumcision Site Capacity and Productivity Assessment Tool (SCPT): An innovative visual management tool to optimize site service delivery

**DOI:** 10.1371/journal.pgph.0000126

**Published:** 2022-01-28

**Authors:** Emmanuel Njeuhmeli, Michel Tchuenche, Marjorie Opuni, Peter Stegman, Matt Hamilton, Steven Forsythe, Felix Nhaduco, Francisco Zita, Nuno Gaspar, Jotamo Come

**Affiliations:** 1 Rollins School of Public Health, Emory University School of Public Health, Atlanta, GA United States of America; 2 Avenir Health, Glastonbury, CT, United States of America; 3 Independent Consultant, Geneva, Switzerland; 4 Jhpiego, Maputo, Mozambique; 5 USAID, Maputo, Mozambique; 6 Ministry of Health, Maputo, Mozambique; Tata Institute of Social Sciences, INDIA

## Abstract

Given constrained funding for HIV, achieving global goals on VMMC scale-up requires that providers improve service delivery operations and use labor and capital inputs as efficiently as possible to produce as many quality VMMCs as feasible. The Voluntary Medical Male Circumcision Site Capacity and Productivity Assessment Tool (SCPT) is an electronic visual management tool developed to help VMMC service providers to understand and improve their site’s performance. The SCPT allows VMMC providers to: 1) track the most important human resources and capital inputs to VMMC service delivery, 2) strategically plan site capacity and targets, and 3) monitor key site-level VMMC service delivery performance indicators. To illustrate a real-world application of the SCPT, we present selected data from two provinces in Mozambique—Manica and Tete, where the SCPT was piloted We looked at the data prior to the introduction of SCPT (October 2014 to August 2016), and during the period when the tool began to be utilized (September 2016 to September 2017). The tool was implemented as part of a broader VMMC site optimization strategy that VMMC implementers in Mozambique put in place to maximize programmatic impact. Routine program data for Manica and Tete from October 2014 to September 2017 showcase the turnaround of the VMMC program that accompanied the implementation of the SCPT together with the other components of the VMMC site optimizatio strategy. From October 2016, there was a dramatic increase in the number of VMMCs performed. The number of fixed service delivery sites providing VMMC services was expanded, and each fixed site extended service delivery by performing VMMCs in outreach sites. Alignment between site targets and the number of VMMCs performed improved from October 2016. Utilization rates stabilized between October 2016 and September 2017, with VMMCs performed closely tracking VMMC site capacity in most sites. The SCPT is designed to address the need for site level data for programmatic decision-making during site planning, implementation, monitoring and evaluation. Deployment of the SCPT can help VMMC providers monitor the performance of VMMC service delivery sites and improve their performance. We recommend use of the customized version of this tool and model to the need of other programs.

## Introduction

Voluntary medical male circumcision (VMMC) is an effective [1–3] and cost-effective [4–6] intervention to reduce female-to-male HIV transmission. In 2007, the World Health Organization (WHO) and the Joint United Nations Programme on HIV/AIDS (UNAIDS) recommended VMMC as an HIV prevention intervention in countries with high HIV prevalence and low levels of male circumcision [7]. WHO and UNAIDS designated the following countries in Eastern and Southern Africa as priority countries for VMMC scale-up: Botswana, Eswatini, Ethiopia, Kenya, Lesotho, Malawi, Mozambique, Namibia, Rwanda, South Africa, Tanzania, Uganda, Zambia, and Zimbabwe [8], with South Sudan added subsequently [9]. The guiding framework for VMMC scale-up had an initial goal of completing 20 million circumcisions between 2011 and 2015, and reaching 80% coverage of circumcision among males ages 15–49 [8]. In 2016, WHO and UNAIDS released revised strategic guidance based on the UNAIDS Fast Track framework [10]. The revised target is to complete 27 million circumcisions by 2021 and to reach circumcision coverage of at least 90% among males aged 10–29 years [11]. Priority countries reported nearly 19 million circumcisions between 2008 and 2017 primarily with support from the US President’s Emergency Plan for AIDS Relief (PEPFAR) and the Global Fund to Fight AIDS, Tuberculosis and Malaria [12, 13]. WHO estimates that this scale-up in VMMCs led to 230,000 HIV infections averted by 2017, with over one million HIV infections projected to be averted by 2030 [12].

The current and future scale-up of VMMC and other HIV interventions must be considered in the context of HIV financing trends. Following more than a decade of significant increases, overall development assistance for HIV in low- and middle-income countries plateaued and has been declining since 2013 [14]. Though domestic HIV funding increased from 2000 to 2017, domestic financing has also declined in the last couple of years [14, 15]. In this context of constrained resources, achieving the ambitious global goals on VMMC scale-up and other HIV targets requires a more focused approach [16, 17]. For VMMC, this means focusing the scale-up of VMMC service provision in a subset of priority subnational locations and on the age groups that will produce the most immediate impact on HIV incidence [18]. It also requires that VMMC providers ensure that sites improve service delivery operations and use labor and capital inputs as efficiently as possible to produce as many quality VMMCs as feasible.

Visual management has been used to improve the performance of production units in health care and other sectors [19]. Visual management tools such as performance walls and dashboards are elements of “Lean”—a quality improvement philosophy which aims to create more value with fewer resources [19–25]. Visual management is closely connected with continuous improvement—a core element of Lean; it captures and visualizes information to provide a shared vision in a production unit of what needs to be improved and the way improvements should be implemented [21]. Most visual management tools used in health care are operational and used primarily to track clinical and health service delivery performance [25–27]. However, some visual management tools include both operational and more strategic aspects to support program planning and monitoring [28]. Though many visual management tools have been manual, an increasing number of them are digital [29, 30].

The Voluntary Medical Male Circumcision Site Capacity and Productivity Assessment Tool (SCPT) is an electronic visual management tool developed to help VMMC providers at site level understand and improve their performance. The tool was initially developed in Microsoft Excel and then converted into a cloud application. The SCPT draws on elements of Lean. The Excel version of the SCPT was developed by USAID Project SOAR, and piloted in Manica and Tete provinces in Mozambique from September 2016 to September 2017. The web-based version of the tool was developed through the USAID supported project Supporting Operational AIDS Research (SOAR). The SCPT is a user-friendly tool capturing and displaying visual information that allows VMMC providers to: 1) track the most important labor and capital inputs to VMMC service delivery, 2) strategically plan site capacity and targets, and 3) monitor key site-level VMMC service delivery performance indicators. To our knowledge, the SCPT is the only tool that allows VMMC implementers to visually monitor inputs and outputs and assess site-level capacity and productivity. It is important to underscore that the primary focus and functionality of the SCPT is at the site level. The data the tool produces is not intended for regional or national aggregation. This article describes the online version of the SCPT. To illustrate a real-world application of the SCPT, we present examples of data from two provinces in Mozambique—Manica and Tete. We present selected data for Manica and Tete from October 2014 to September 2017 to highlight the substantial increases in VMMC coverage made over that period, and describe the role played by the SCPT in the broader VMMC site optimization strategy put in place in Mozambique from September 2016. In fact, VMMC procedures were decentralized and performed from outreach sites and /or referral to fixed sites. That is, while the number of fixed service delivery sites providing VMMC services in Manica and Tete was expanded, and each fixed site extended service delivery by performing VMMCs in outreach sites.

The VMMC program in Mozambique was implemented since 2010 in the provinces with high HIV Prevalence and low male circumcision prevalence. Manica and Tete were not the only Provinces implementing VMMC. Therefore, one cannot define a non-intervention comparison because the improvement plan was designed to improve the program in Manica and Tete and not to compare Manica and Tete program performance with that of other Provinces. By 2015, the VMMC program in these two provinces were the less performing with less than 15% achievement vs target. When the SCPT was introduced as part of a comprehensive improvement strategy in 2016, by middle of 2017, the VMMC program in Manica and Tete has become the most performing program in Mozambique. It is important to highlight that the program was implemented in most of these Provinces by the same Implementing partner. However, it is not possible to attribute the site improvement and performance to one single tool or interventions because the VMMC program benefitted from a baseline assessment, the continuous quality improvement to determine performance against every single standard.

## Methods

### Ethical considerations

This study did not entail research involving individual human participants and/or the collection of medical data for individual participants. Furthermore, interviews of patients were not conducted as part of this research. To run the SCPT requires only routine voluntary medical male circumcision program data. All program data were obtained in aggregate and made available to the research team with no names or other personal identifiers for individual patients. Therefore, it was determined by Project SOAR, Population Council and Avenir Health that ethical approval was not necessary.

The SCPT is a digital visual management tool for VMMC service providers initially developed in Microsoft Excel. To take advantage of multi-user capabilities, the SCPT was converted into a cloud application hosted on Microsoft Azure. The system architecture was built with Embarcadero Delphi backed with Excel/CSV for data storage. The front end was developed with JavaScript/React using Material-UI. The current version of the SCPT can be accessed here: http://www.vmmcipt.org//, a VMMC data for decision-making toolkit portal managed by Avenir Health. Once the SCPT is accessed, resources including a detailed user’s guide and data management guidelines can be found. We describe below the current version of the SCPT and specify any important differences that exist between the online and Excel versions of the tool.

### SCPT inputs

The intention of the SCPT is to capture and visually present all relevant and available data in one place to enhance the ability of site managers to strategically plan VMMC service delivery. Table 1 displays user inputs into the tool. These include data on site characteristics (e.g., site location, site ownership, VMMC service delivery mode, task sharing and task shifting status, etc.), site VMMC targets, site personnel working on VMMC and surgical bed availability, and site performance (e.g. number of clients reached by mobilizers by age group, number of VMMCs performed by age group, number of post-surgery follow-ups, number of adverse events, etc.). The SCPT captures data by month. Data can be entered into the tool by country partners with permission to enter facility data—usually monitoring and evaluation officers in PEPFAR implementing partner organizations. The SCPT is a tool focused for use at the site level, and data can be entered into the tool by site managers, data managers whether at the Ministry of Health or by an implementing partner depending on how the data process (gathering, cleaning is organized and who has the responsibility to enter it into the tool)—whenever cleaned site-level data are available. The tool offers both online and offline data capture and upload functionalities to accommodate those sites where internet access is poor or restricted.

**Table 1:** SCPT inputs.

Input categories	Inputs
**Site characteristics**	
	Site name
	Health zone
	Province/region
	Rural/urban
	Site ownership (public/private/nongovernmental organization)
	DREAMS status (DREAMS/non-DREAMS)
	Implementing US government agency
	Implementing partner
	Service delivery mode (fixed/mobile/outreach)
	PEPFAR category (attained/scale-up aggressive/scale-up saturation)
	VMMC category (scale-up/sustainability/saturated)
	Task sharing status (true/false)
	Task shifting status (true/false)
	Site opening date
**Site VMMC target**	
	# VMMCs site aims to achieve
**Personnel & beds**	
	# VMMC providers
	# trained assistants
	# other nurses
	# mobilizers
	# counselors
	# VMMC surgical beds
**Site performance**	
	# males reached by mobilizers by age group
	# males seeking VMMC services by age group
	# males reached by information source (TV, radio, friend, family, partner, mobilizers, community leaders, posters, health workers)
	# days of operation
	# VMMCs performed by age group
	# post-surgery follow-ups (48 hours, 7 days, 6 weeks)
	# adverse events (mild, moderate, severe)
	# STI (VMMC clients only)
	# STI treated and circumcised (VMMC clients only)
	# HIV tests performed (VMMC clients only)
	# HIV+ referred for treatment (VMMC clients only)

### SCPT outputs

The SCPT allows users to display all the data entered in the combinations he or she chooses. SCPT users can elect to display site level data or aggregated data for a health zone, province, or region. Users can choose the time frame and format (graphs or tables) in which the data is to be displayed. SCPT users can also choose the time interval for the data display—month, quarter, half-year, or year. Besides displaying the data entered in various ways, the tool can also calculate many permutations of the information entered. For example, while users enter the number of VMMCs performed per month, the tool can generate the average number of VMMCs performed daily in a given time period calculated as the number of VMMCs performed divided by the number of days of operation during the time period of interest. In addition to facilitating the collation and reorganization of information, the SCPT also calculates several indicators using the information entered by users. These include: current site capacity, optimum site capacity, additional staff needed for optimum site operation, utilization rate, and productivity gain/loss. We describe each of these below.

Current and optimum site capacity are based on existing international guidelines on optimized client flow and quality VMMC service delivery [31–33], research on surgical efficiencies [34], and VMMC cost drivers [35]. Current site capacity is defined as the number of VMMCs that a site should be able to perform over a specified time period given available VMMC service delivery inputs including VMMC surgical beds, VMMC providers (who perform the circumcision), trained assistants (who perform the pre-operative assessment), nurses (who provide post-operative care), counsellors (who provide VMMC counseling, HTC, and client education), and community mobilizers (who assist with community demand generation and client post-operative follow-up). Optimum site capacity is defined as the number of circumcisions that could be performed during a set time period given the efficient use of VMMC surgical beds in a site along with optimized staffing arrangements. To calculate site capacity, the SCPT assumes that each procedure is undertaken in 30 minutes. The tool differentiates between sites with and without task shifting (the delegation of the entire VMMC procedure from a physician to a trained nurse or clinical officer) and task sharing (the delegation of some parts of the VMMC procedure to a trained nurse or clinical officer with the highest-skill steps undertaken by a physician) [36]. The tool estimates current capacity by taking into account staff currently working in the facility, while for optimum capacity, the tool takes a normative approach by applying the total number of staff capable of providing VMMC services given the number of surgical beds in the facility. The Excel version of the tool calculated current daily site capacity as the number of VMMC providers times 15 and optimum daily site capacity as the number of surgical beds times 15.

The utilization rate is the ratio of VMMCs performed to current site capacity. If a site performs 15 VMMCs per day and its current daily capacity is 15, then its daily utilization rate would be 15/15, or 1. If a site conducts 10 VMMCs per day and its current daily capacity is 15, its utilization rate would be 10/15, or 0.67 meaning that the site is under-used. On the other hand, if a site conducts 19 circumcisions per day and its current daily capacity is 15, its utilization rate would be 19/15, or 1.27, meaning that the site is over-used (perhaps a site requires overtime or needs to employ temporary workers to meet the demand). Both under-used and over-used sites are cause for concern. Under-utilized sites are inefficient—additional VMMCs could be performed given the staff and surgical beds available. Over-utilized sites may be stretching their resources too thinly or may be cutting operational corners to meet excess demand or overly ambitious service delivery targets. Monitoring of service quality is critical in all sites, but over-used sites should be monitored especially closely to ensure that efficiency is accompanied by service quality.

The SCPT also provides program and site managers with indications of productivity gains and losses by presenting the difference between the number of VMMCs performed and the number of VMMCs the facility could have performed if operating at its optimum capacity. For example, if the number of daily VMMCs performed is 10 and the optimum daily site capacity is 10, productivity would be 10–10 = 0 or, if 10 circumcisions are performed a day and the optimum daily site capacity is 15, the productivity loss would be 10–15 = -5. On the other hand, if the number of daily VMMCs performed is 18 and the optimum daily site capacity is 15, the productivity gain would be 18–15 = 3. Both productivity losses and gains need to be explored. Productivity losses are driven by mismatches between the staff allocated to a site and the number of VMMC surgical beds in the facility. Productivity gains are driven by over-utilization in sites where current capacity has been optimized.

## Results

As a case study of a real-world application of the SCPT, we present below examples of data from Manica and Tete provinces in Mozambique—where the SCPT was piloted from September 2016 to September 2017. We present selected data from October 2014 to September 2017 to showcase the improvements in VMMC service delivery the program made over the period.

**Fig 1:**
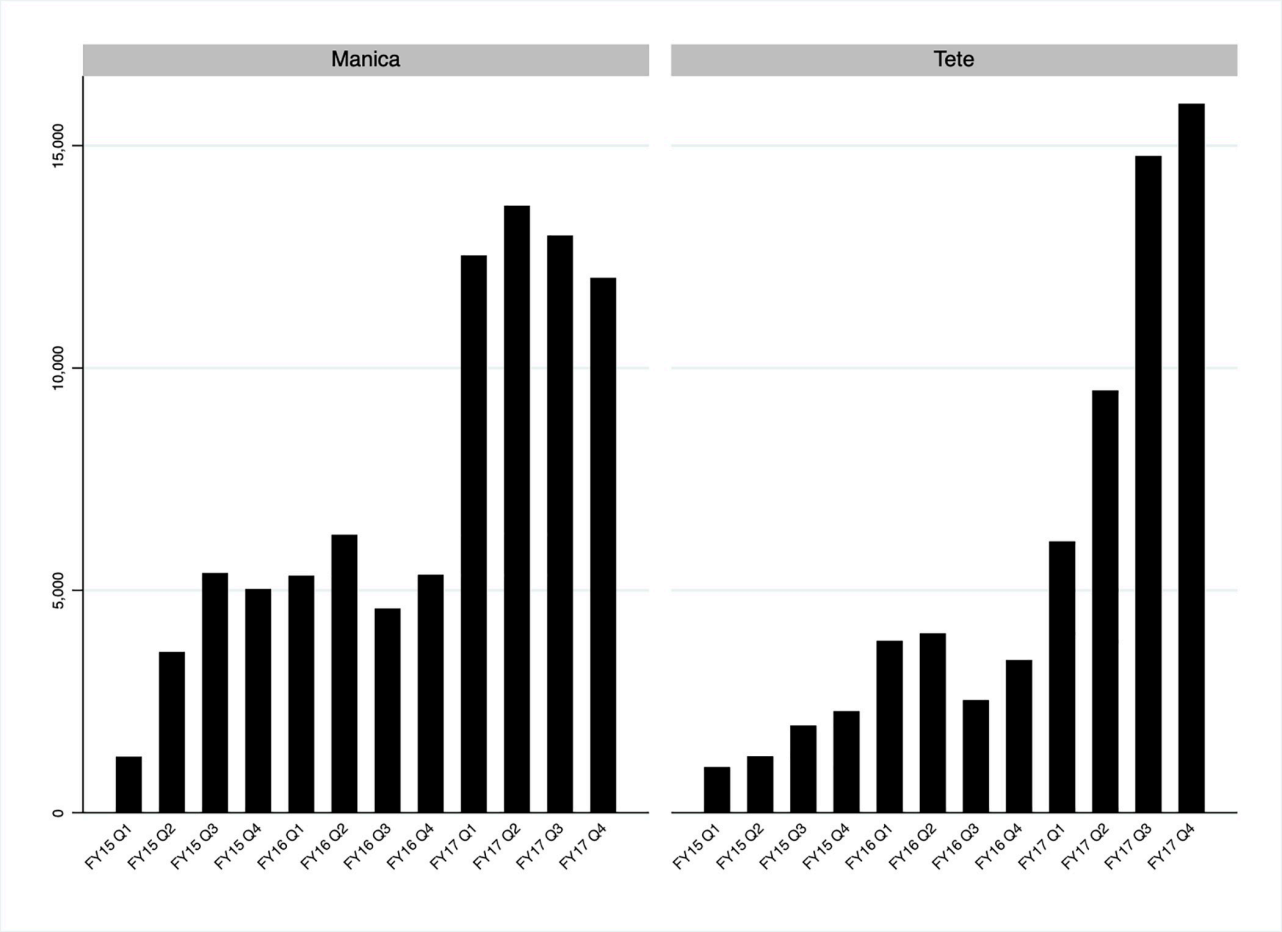
Voluntary medical male circumcisions (VMMCs) performed in Manica and Tete, Mozambique FY15 Q1- FY17 Q4. Fiscal year (FY) refers to the fiscal year of the United States Government which begins on October 1 and ends on September 30. The fiscal year is designated by the calendar year in which it ends–e.g., fiscal year 2015 begins on October 1, 2014, and ends on September 30, 2015. The four quarters of each fiscal year (Q1-Q4) are October-December, January-March, April-June, and July-September.

**Fig 2:**
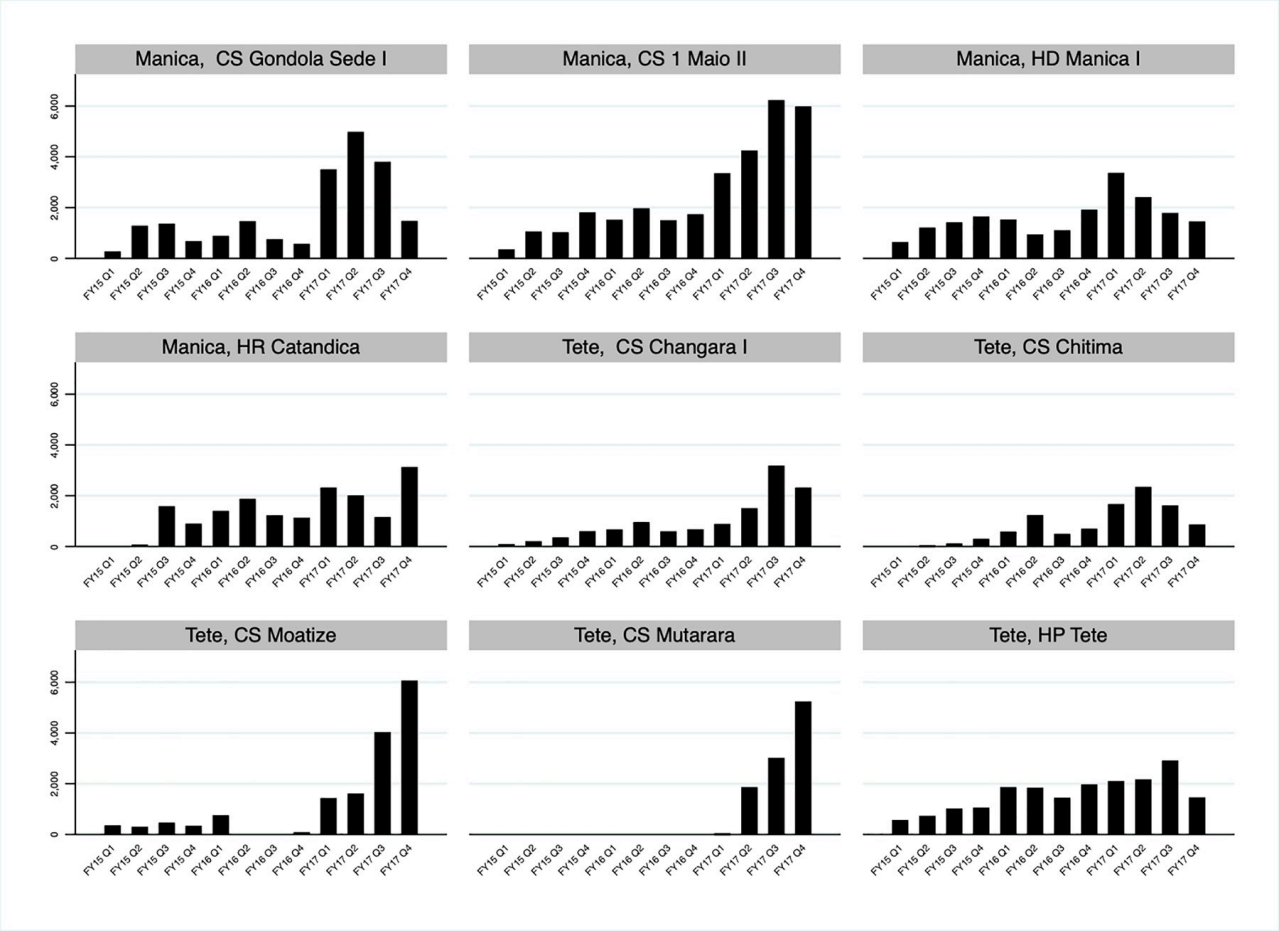
Voluntary medical male circumcisions (VMMCs) performed in Manica and Tete, Mozambique FY15 Q1- FY17 Q4 by fixed service delivery site.

Over the three-year period from October 2014 to September 2017, the total number of VMMCs performed increased from 1,259 to 12,029 in Manica and from 1,024 to 15,943 in Tete (Fig 1). This is in line with the steady increases in the numbers of VMMCs performed over time in most of the nine fixed service delivery sites performing VMMCs in the two provinces: CS Gondola Sede I, CS 1 Maio II, HD Manica I, and HR Catandica in Manica; and CS Changara I, CS Chitima, CS Moatize, CS Mutarara, and HP Tete in Tete (Fig 2).

**Fig 3:**
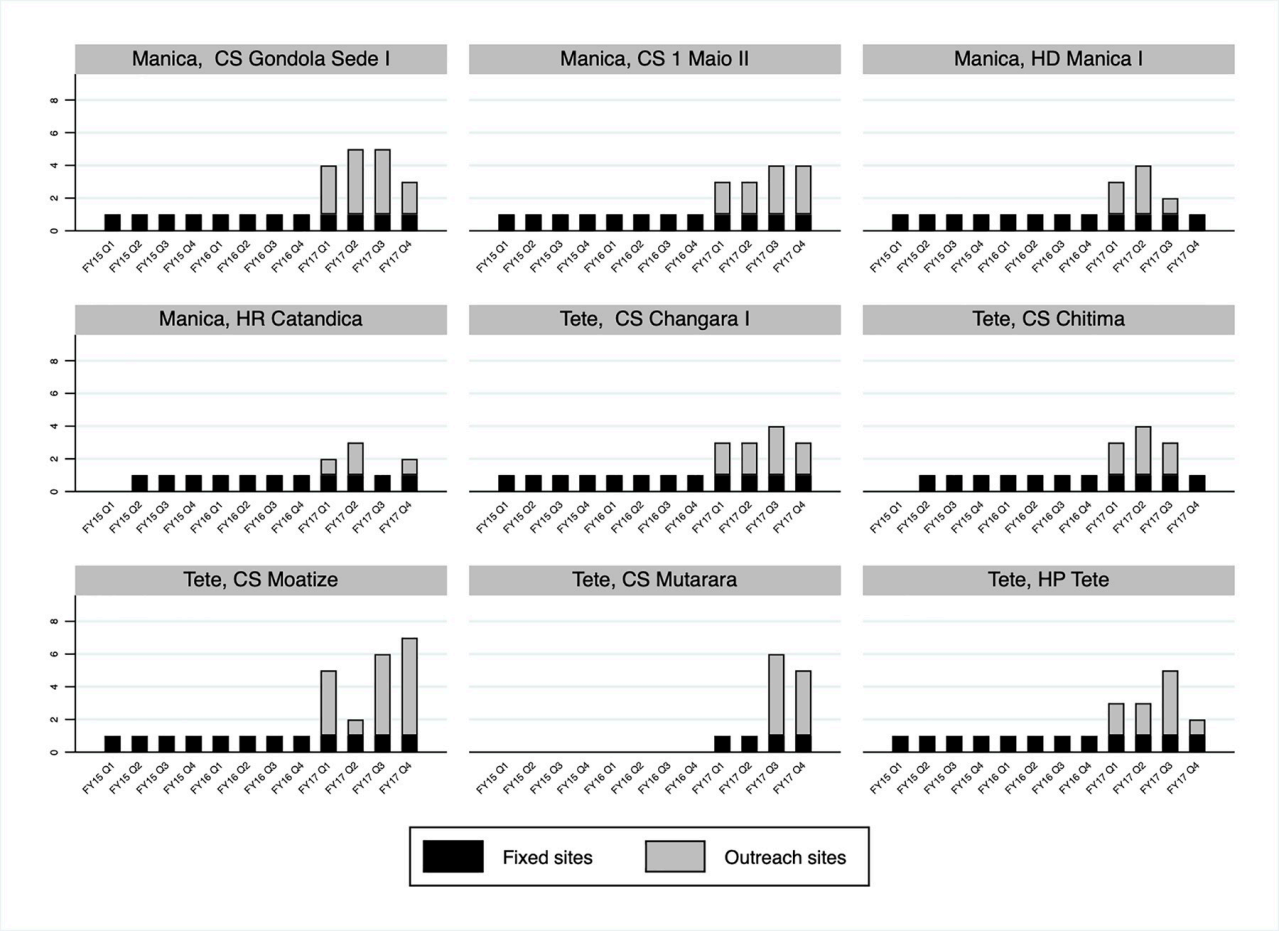
Numbers of fixed and outreach service delivery sites performing voluntary medical male circumcisions (VMMCs) in Manica and Tete, Mozambique FY15 Q1- FY17 Q4 by fixed service delivery site.

Several logistic factors including the service delivery traking using the SCPT contributed to this increase in numbers of VMMCs performed over time in Manica and Tete. The number of fixed service delivery sites providing VMMC services was expanded over time with CS Mutarara beginning to perform VMMCs in October 2016 (Fig 3). In addition, from October 2016, each fixed site expanded service delivery by performing VMMCs in outreach sites—sites located away from a fixed site but overseen and managed by staff in the fixed site (Fig 3). Between 18 and 27 outreach sites were affiliated with the nine fixed service delivery sites in Manica and Tete in the four quarters between October 2016 and September 2017.

**Fig 4:**
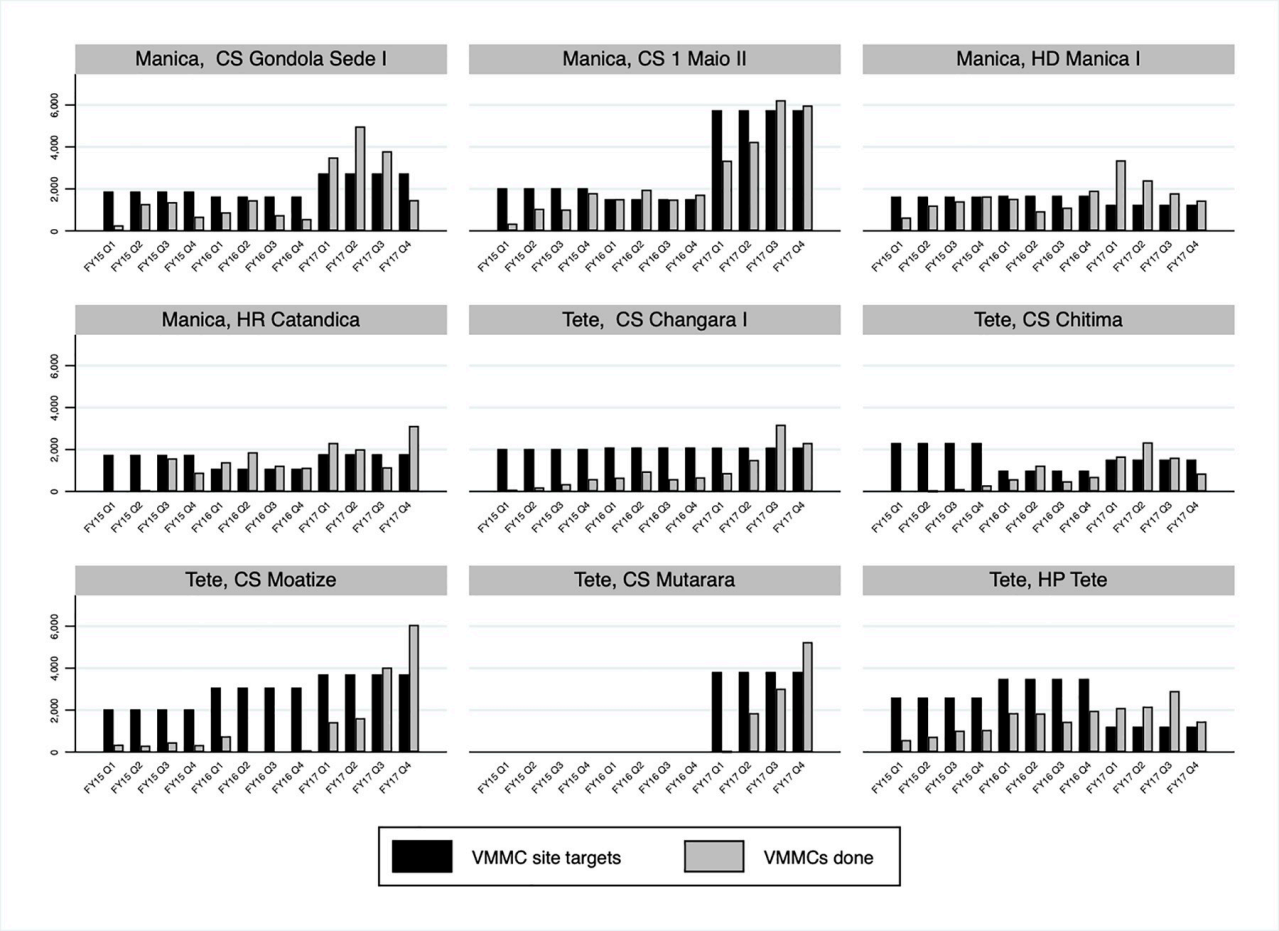
Voluntary medical male circumcisions (VMMCs) site targets and VMMCs performed in Manica and Tete, Mozambique FY15 Q1- FY17 Q4 by fixed service delivery site.

**Fig 5:**
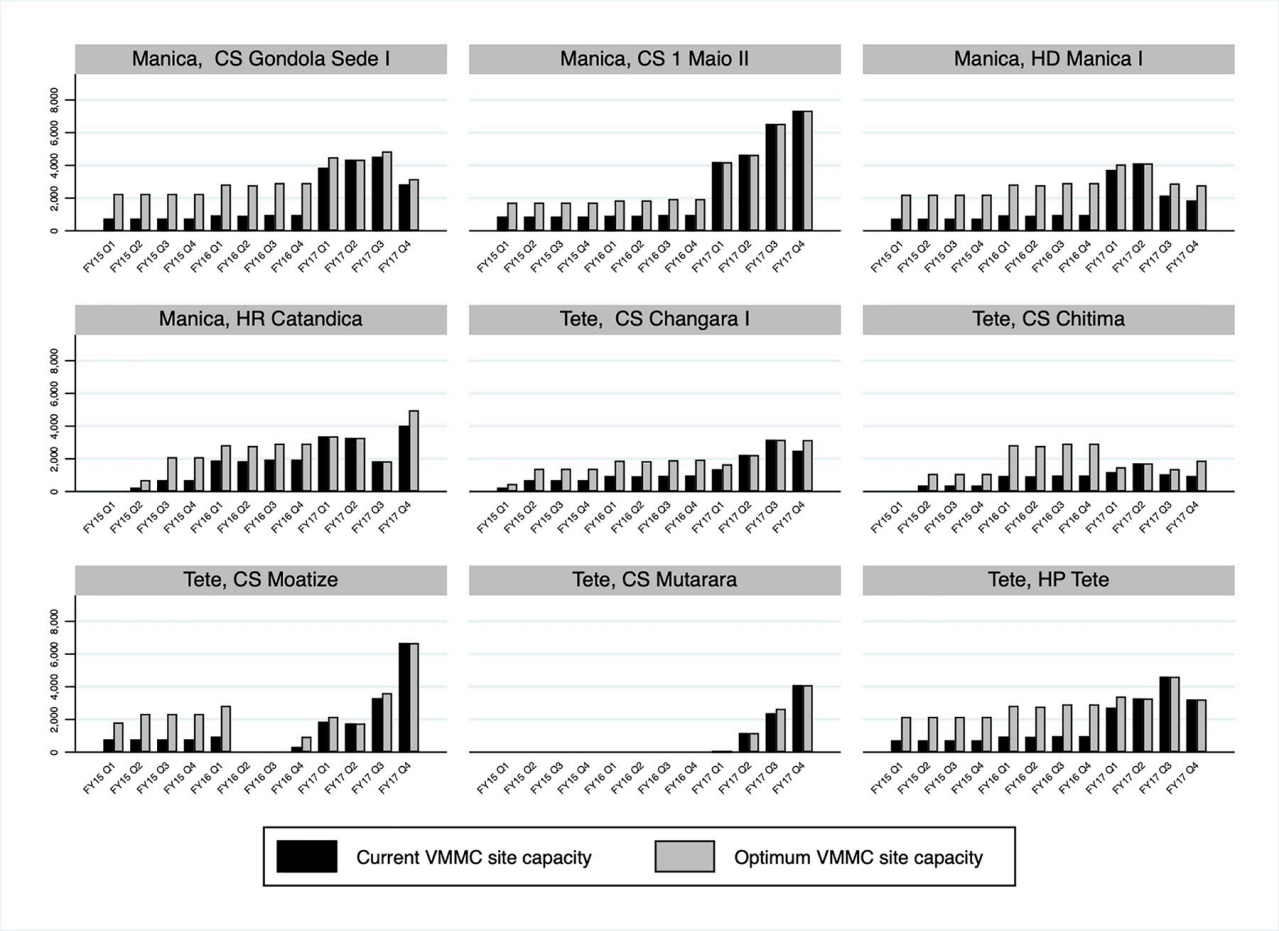
Current voluntary medical male circumcisions (VMMCs) site targets and optimum VMMC site capacity in Manica and Tete, Mozambique FY15 Q1- FY17 Q4 by fixed service delivery site.

**Fig 6:**
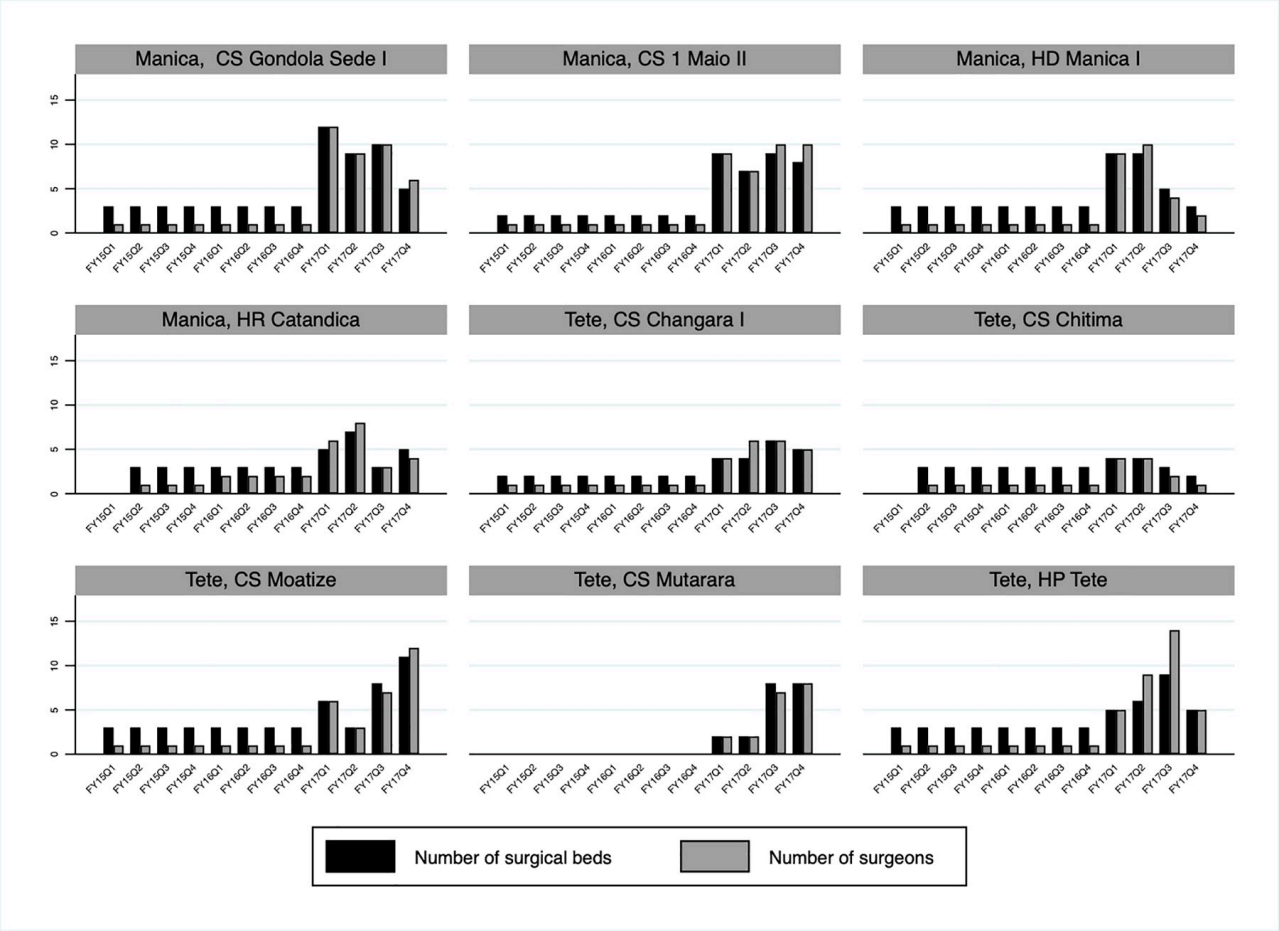
Numbers of surgical beds and surgeons in Manica and Tete, Mozambique FY15 Q1- FY17 Q4 by fixed service delivery site.

Assessment of service delivery site indicators over time also shows that several additional site-level implementation changes accompanied the important increase in VMMCs performed in Manica and Tete between October 2014 and September 2017. Alignment between VMMC site targets and the number of VMMCs performed improved, with the number of VMMCs performed much more likely to equal or surpass VMMC site targets at the end of the period (Fig 4). In addition, significant improvements in current VMMC site capacity were made based on the outcome of the pilot implementation of the tool and quality assurance (the latter being conducted separately, but around the same time period of the pilot), with current VMMC site capacity equal or very close to optimum VMMC site capacity in most sites at the end of the period (Fig 5). This was underpinned by the increase in the number of surgical beds and the number of surgeons in Manica and Tete over time, and as importantly, with an improved alignment between the two in most sites (Fig 6).

**Fig 7:**
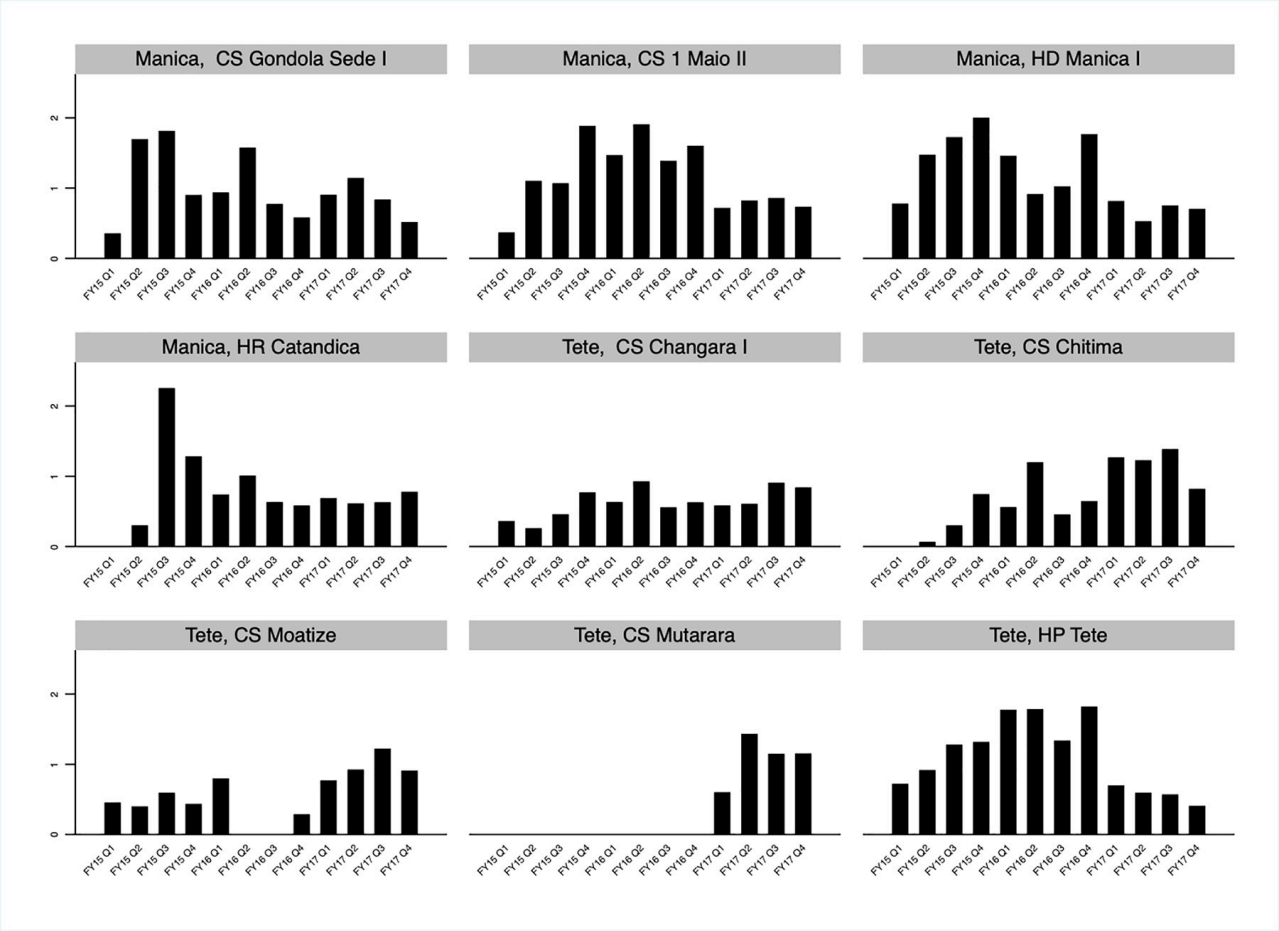
Utilization rate for voluntary medical male circumcisions (VMMCs) service delivery sites in Manica and Tete, Mozambique FY15 Q1- FY17 Q4 by fixed service delivery site. Utilization rates are calculated for fixed service delivery sites and associated outreach sites. The utilization rate is the ratio of VMMCs performed to current VMMC site capacity.

The utilization rates—the ratio of VMMCs performed to current VMMC site capacity—in service delivery sites providing VMMC services in Manica and Tete were low in October 2014 (Fig 7). There was a great deal of fluctuation in utilization over time in most sites between October 2014 and September 2016, with some sites under-utilized in some quarters and others severely over-utilized. Utilization rates stabilized between October 2016 and September 2017, with VMMCs performed closely tracking VMMC site capacity each quarter in most sites.

**Fig 8:**
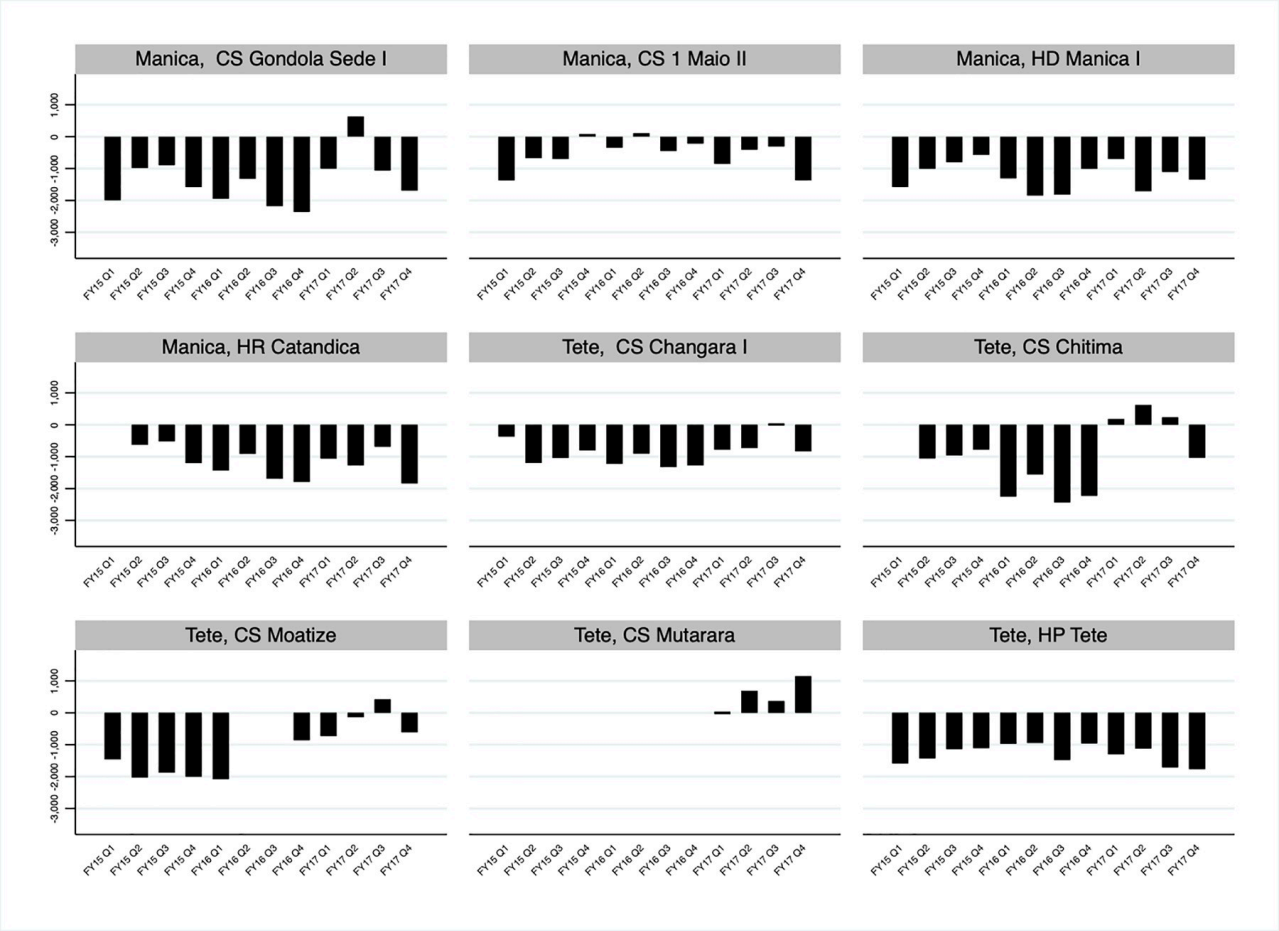
Productivity gains and losses for fixed voluntary medical male circumcisions (VMMCs) service delivery sites in Manica and Tete, Mozambique FY15 Q1- FY17 Q4. Productivity gains and losses are calculated for fixed service delivery sites and associated outreach sites. The productivity gain/loss is the number of VMMCs performed minus the number of VMMCs the site could have performed at optimum site capacity.

Productivity gains/losses—the number of VMMCs performed minus the number of VMMCs the site could have performed at optimum capacity—in VMMC service delivery sites in Manica and Tete varied across sites and by quarter between October 2014 and September 2017, but they were usually negative, meaning they did not perform the number of circumcisions possible based on the capacity of the site (Fig 8). Comparing site productivity (Fig 8) with site utilization (Fig 7) over time highlights that even though site utilization stabilized around one between October 2016 and September 2017, most sites could still have made substantial efficiency gains by better aligning VMMC staff and VMMC surgical beds.

**Fig 9:**
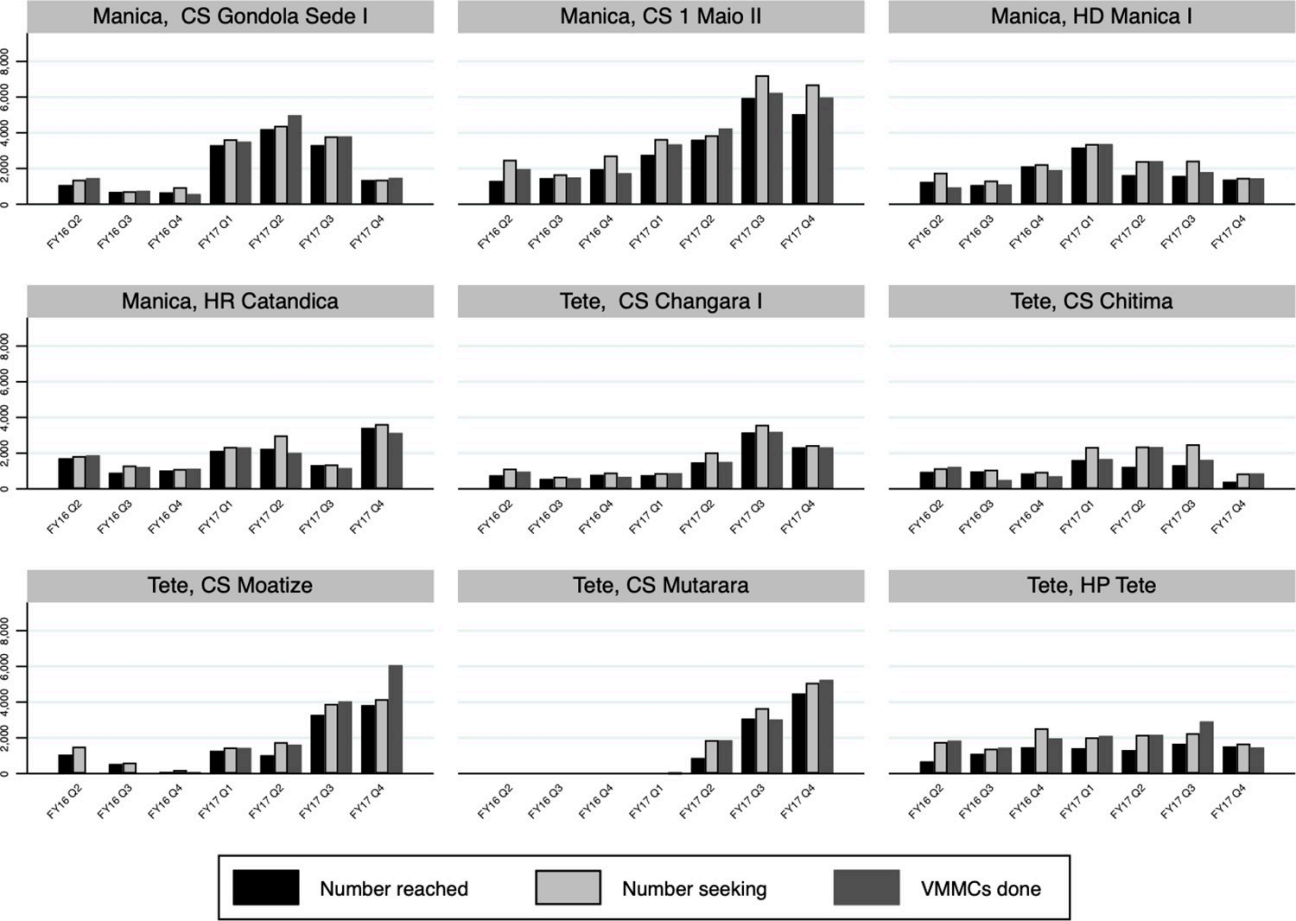
Voluntary medical male circumcisions (VMMCs) cascade in Manica and Tete, Mozambique FY16 Q2- FY17 Q4 by fixed service delivery site. Number reached—number of people reached by VMMC services; number seeking—number of people seeking VMMC services; VMMCs done—number of VMMCs performed. Numbers are totals for fixed service delivery sites and associated outreach sites.

The VMMC cascade includes the number of people reached by VMMC demand creation services, the number of people seeking VMMC services, and the number of VMMCs performed. Fig 9 displays the VMMC cascade for service delivery sites in Manica and Tete for the time period for which data are available—January 2016 to September 2017. Increases in the numbers of people reached by demand creation services, the numbers of people seeking VMMC services, and the numbers of VMMCs performed over time are observed in half the sites. In all sites, during most quarters, however, the number of people seeking VMMC services was greater than the number of people reached.

## Discussion

To achieve the ambitious global goals for the scale-up of VMMC scale-up and other HIV interventions in the context of constrained resources, all providers must improve service delivery operations and use labor and capital inputs as efficiently as possible. The SCPT is a visual management tool that was developed to help providers monitor the performance of VMMC service delivery sites and to strategically improve that performance to ensure that sites produce as many quality VMMCs as feasible. Specifically, the SCPT allows VMMC implementers to:

- Track the key labor and capital inputs to VMMC service delivery;
- Monitor the numbers of VMMCs performed;
- Estimate current and optimum site service delivery capacity;
- Compare VMMCs performed with site targets and current and optimum site capacity;
- Estimate additional staffing needs to achieve optimum site capacity;
- Monitor the reach and impact of VMMC demand creation efforts;
- Compare VMMCs done with number of people reached by VMMC demand creation efforts and number of people seeking services;
- Track circumcision follow-up visits and number of adverse events;
- Track uptake of HIV testing, and STI services;
- Estimate site utilization and site productivity gains/losses.

An examination of Manica and Tete provinces in Mozambique, where there was a major strategic restructuring of the program underway and where the SCPT was piloted from September 2016 to September 2017, provides insights into the information that can be obtained from the tool and its potential impact. Although VMMC services were available in Manica and Tete provinces from 2012, coverage remained extremely low by end of 2014. Some progress was made over the next year although coverage remained low. Graphical displays of data using the SCPT were initially analyzed by VMMC implementers in September 2015. However, it took some time to get all stakeholders on board, underscoring the importance of early involvement of end-users in the adoption of the SCPT. Initially, providers thought that the discrepancies between number of VMMCs done and site targets was the result of overly ambitious targets and not differences between number of VMMCs done and site capacity. VMMC implementers in Mozambique began using a VMMC site optimization strategy from September 2016. This strategy was developed based on the significant gaps identified between the number of VMMCs done per site and site capacity given available inputs as well as optimum site capacity given altered inputs. In addition to using the SCPT, the VMMC site optimization strategy involved monthly brainstorming by implementers, development of site-specific targets, and monitoring and regular updating of targets, and continuous service delivery quality improvement.

From October 2016, there was a dramatic increase in the number of VMMCs performed in Manica and Tete provinces. The number of fixed service delivery sites providing VMMC services was expanded. In addition, each fixed site extended service delivery by performing VMMCs in outreach sites. This led to significant improvements with current VMMC site capacity increasing and in some cases very close to the site optimum in most sites by the end FY 2017. Importantly, the inclusion of outreach sites also improved access by bringing services closer to harder to reach and lower-income clients. Fixed sites are often located in urban and peri-urban locations and out-of-pocket travel expenses associated with accessing VMMC services can be quite high and lost time and associated income can also be an issue for some [37].

Based on the information site and/or program managers obtained through tracking service delivery using the SCPT, alignment between VMMC site targets and the number of VMMCs performed improved from October 2016, with the number of VMMCs performed much more likely to equal or surpass VMMC site targets at the end of the period. Utilization rates stabilized between October 2016 and September 2017, with VMMCs performed closely tracking VMMC site capacity each quarter in most sites. Both under- and over- utilization were problematic. Factors that influence utilization in addition to site level planning include seasonality, quality and reach of demand creation activities, client access, quality of in-service communication, and client satisfaction with service delivery (e.g. mixing younger and older men may result in older men declining services) [31, 32]. All of these factors were identified as issues to be dealt with in order to improve the performance of VMMC service delivery sites in Manica and Tete. Monthly reviews of SCPT graphical displays by implementers allowed providers to monitor and adjust the services provided on an ongoing basis.

Increases in the numbers of people reached by VMMC services, the numbers of people seeking VMMC services, and the numbers of VMMCs performed over time were observed in half the sites. The fact that the number of people seeking VMMC services was greater than the number of people reached in all sites during most quarters underscores the importance of in-service communications and the potential for strengthening demand creation approaches implemented in Manica and Tete [38, 39].

Envisioned as a planning tool as well, one of the strengths of the SCPT is that VMMC providers can use it to visually represent data on their site’s performance over time. Together with regular reviews of these graphical displays and using the information to recalibrate service delivery, implementers can continuously improve their performance. The power of visual management tools and the role that they can play in improving health facility-level performance is well documented [40–43]. Moreover, in addition to visually representing available data in numerous different ways, the SCPT also calculates estimates based on the information entered that is useful to VMMC implementers including current and optimum site capacity, site utilization, and site productivity. Another strength of the SCPT is that it is easy to use both in terms of entering and extracting data. It includes data that are easily available and routinely collected by VMMC service delivery sites and allow for the monitoring of productivity and quality.

Lack of data for site level decision making prompted the development of the SCPT. Before the tool, the site managers had not relevant information on how plan for demand, capacity, quality, and efficiency of the site, focusing only on the use of the achievement vs target as indicator to discuss performance and improvement. With the SCPT, the site managers were able to have information related to target vs capacity vs demand, performance, quality, and efficiency. Being able to precisely plan the number of staff for example base of the site target allow the site manager to bring the site capacity to the level of the target, therefore given them greater opportunities to provide services to all clients who needed it. However, it is not possible to attribute the site improvement and performance to one single tool or interventions. The program benefitted from a baseline assessment (continuous quality improvement–CQI) to determine performance against every single standard.

As with all monitoring, planning, and modeling tools of this kind, the utility of the SCPT is limited by the amount and quality of data that are entered into it. Even though the data included in the SCPT are routinely collected by sites receiving PEPFAR funding, the quality and level of completeness of the data varies. Another limitation of the SCPT is that it is difficult to compare across sites and identify in what ways well performing sites differ from less well-performing sites. Benchmarking—where performance across sites is compared and top performers are identified and best practices are detected so they can be emulated by peers—is a tool for performance improvement long used in health care and other sectors [44]. An additional limitation of the tool is that it does not include any information on VMMC service costs or on VMMC expenditures by site. In part this is because cost information is not currently tracked by sites, so it is not routinely available. Previous work identified substantial differences in unit costs of VMMC services within and across countries, suggesting that substantial technical efficiency gains could be made in VMMC service delivery in many sites [35, 45].

Because the SCPT was built primarily as a site level tool, its principal aim is to pull all relevant data into one repository to support site managers and staff to monitor achievement of VMMC targets and ensure that the resources available at the facility are used to maximize service delivery. However, the tool is also useful for managers above the site level (district, regional, national, etc.) as they can use the SCPT to obtain insights into program implementation and performance for larger administrative areas. Current discussions about the continued use of the SCPT revolve around identifying the appropriate place for the tool within existing health information systems, such as the District Health Information Software 2 (DHIS2) used in many low and middle-income countries. Given the specificity of the tool both in terms of its programmatic focus and implementation level, Ministries of Health are exploring what technical options may exist to maintain the tool’s use at site level while being able to both pull and push data to the DHIS2. Another option would be to customize the DHIS2 to capture the same inputs and provide same dashboard as the SCPT.

While the application of the SCPT in Manica and Tete provinces in Mozambique from September 2016 to September 2017 was not the sole factor underlying the significant scale-up of VMMC service delivery, the use of the tool as part of a broader site optimization strategy showed encouraging results. Workshops and webinars on SCPT have been conducted in Eswatini, Kenya, Lesotho, Malawi, Namibia, South Africa, Tanzania, Uganda, and Zimbabwe in addition to Mozambique. Other countries should consider using the SCPT to improve the performance of VMMC service delivery sites and ensure that they use labor and capital inputs as efficiently as possible to produce as many quality VMMCs as feasible.

Finally, the SCPT is a programmatic and implementation tool to improve efficiency and performance of VMMC with the potential strength of using routine data. The operational capabilities of the tool could be adapted and applied to other programmatic areas to drive performance improvements.
